# Integrative single-cell and bulk transcriptomics define polyamine-associated cell states in acute myeloid leukemia and implicate CCT6A in polyamine homeostasis

**DOI:** 10.3389/fimmu.2026.1914578

**Published:** 2026-09-08

**Authors:** Aimei Feng, Xiaohe Chen, Fan Chen, Xiaowu Wang, Mengjia Peng, Jue Zeng, Ming Chen

**Affiliations:** 1 Department of Hematology, The Third Affiliated Hospital of Wenzhou Medical University, Ruian, China; 2 Department of Blood Transfusion, The Third Affiliated Hospital of Wenzhou Medical University, Ruian, China; 3 Department of Burn and Skin Repair Surgery, The Third Affiliated Hospital of Wenzhou Medical University, Ruian, China; 4 Department of Obstetrics and Gynecology, The Third Affiliated Hospital of Wenzhou Medical University, Ruian, China

**Keywords:** acute myeloid leukemia, machine learning, polyamine metabolism, prognostic signature, random survival forest, single-cell RNA sequencing, tumor microenvironment

## Abstract

**Background:**

Polyamine metabolism is an important metabolic dependency in cancer, but its cellular organization and clinical relevance in acute myeloid leukemia (AML) remain unclear.

**Methods:**

We integrated publicly available single-cell RNA sequencing (scRNA-seq) data from 41 AML samples (198,846 cells) and bulk transcriptomic profiles from 712 AML patients across independent cohorts. Polyamine activity was quantified using a 76-gene signature. Consensus clustering and machine-learning algorithms were used for molecular classification and prognostic modeling. Immune features and gene dependency were further evaluated. CCT6A was further validated by quantitative real-time PCR (qPCR), small interfering RNA (siRNA) silencing, cell viability and apoptosis assays, Western blotting, intracellular polyamine and ODC1 activity measurements, and spermidine rescue.

**Results:**

Polyamine activity was preferentially enriched in AML blasts and associated with increased stemness, proliferation, and DNA damage response, together with impaired differentiation. Two molecular subtypes with distinct metabolic and immune features were identified. The Random Survival Forest model effectively stratified patients into prognostic risk groups, achieving external-validation area under the curve (AUC) values up to 0.774. High-risk patients exhibited distinct immune and stromal microenvironmental features. CCT6A was overexpressed in AML samples, and its silencing suppressed cell viability, promoted apoptosis, and reduced ODC1 activity and intracellular polyamine levels. Importantly, exogenous spermidine substantially restored cell viability following CCT6A knockdown.

**Conclusion:**

Polyamine-active AML blast states have prognostic and immune relevance, while CCT6A is functionally linked to AML cell fitness and polyamine homeostasis.

## 1 Introduction

Acute myeloid leukemia (AML) is a genetically and biologically heterogeneous hematologic malignancy characterized by the clonal expansion of poorly differentiated myeloid precursors. Despite advances in molecular classification, risk stratification, and treatment, long-term survival remains below 30%, largely because of relapse and therapy resistance (1, 2). Importantly, genetic alterations alone do not fully explain the marked variability in disease behavior and therapeutic response. Increasing evidence indicates that metabolic reprogramming and interactions within the bone marrow microenvironment are important non-genetic determinants of leukemic stem cell maintenance, immune evasion, and treatment resistance (3–5). Therefore, identifying metabolic programs associated with aggressive leukemic states may complement existing molecular classification and improve biologically informed patient stratification.

Polyamine metabolism represents a particularly relevant metabolic axis in this context. Polyamines, mainly putrescine, spermidine, and spermine, are tightly regulated metabolites required for cell growth, proliferation, translation, and epigenetic regulation (6–8). Dysregulated polyamine homeostasis has been associated with tumor progression and adverse clinical phenotypes across multiple cancer types (9–11). In AML, spermidine is enriched in primary human leukemic stem cells, and disruption of polyamine-related metabolic processes impairs leukemic stem cell self-renewal and engraftment (12). Moreover, the context-dependent role of SAT1 in acute leukemias suggests that polyamine metabolism is not uniformly activated but may instead undergo lineage- and state-specific rewiring (13). These observations raise the possibility that polyamine metabolism contributes to AML heterogeneity by supporting distinct malignant cell states and their interactions with the surrounding microenvironment.

However, the cellular organization and clinical relevance of polyamine metabolism in AML remain incompletely defined. It is unclear which bone marrow cell populations preferentially engage this pathway, whether polyamine-active AML blasts display distinct stemness and functional programs, and how these metabolic states relate to immune and stromal remodeling. Such heterogeneity cannot be adequately resolved by bulk transcriptomic profiling alone. Single-cell RNA sequencing enables cell type- and state-specific characterization of transcriptional programs (14–16), whereas integration with spatial and bulk transcriptomic data can further connect these cellular states with tissue organization, immune contexture, and clinical outcomes (9, 17, 18). A multi-layered strategy may therefore help link cellular metabolic heterogeneity to clinically relevant AML subtypes and patient risk.

Building on our previous work showing that ribosome biogenesis-related states capture clinically relevant AML heterogeneity across single-cell and bulk transcriptomic levels (19), we asked whether polyamine metabolism represents a distinct and functionally testable metabolic axis that can further resolve AML heterogeneity through blast-state, spatial, and perturbation–rescue analyses. We hypothesized that polyamine metabolism defines specific malignant cell states in AML and that genes associated with these states could capture clinically relevant biological heterogeneity. To test this hypothesis, we integrated single-cell, spatial, and bulk transcriptomic data to map polyamine metabolism across the AML bone marrow microenvironment, characterize its associations with stemness, functional programs, and intercellular communication, and identify polyamine-associated molecular subtypes. We further developed and externally validated a machine-learning-based prognostic model and examined its relationships with immune features, predicted drug sensitivity, gene dependency, and spatial localization. Finally, the prioritized candidate CCT6A was experimentally evaluated through gene silencing, polyamine profiling, and rescue assays. Together, this study establishes a polyamine-centered framework linking metabolic heterogeneity to AML risk stratification and candidate functional vulnerabilities.

## 2 Methods

### 2.1 Collection and preprocessing of single-cell and spatial transcriptomic data

Single-cell RNA sequencing (scRNA-seq) data from 41 AML samples were sourced from GSE185381 (n = 20) (20), GSE116256 (n = 16) (21), and zenodo.3345981 (n = 5) (22), and were analyzed using Seurat v5.3.0 in R v4.4.1. Cells were excluded if they had a mitochondrial read proportion >20%, unique molecular identifier (UMI) counts <200 or >80,000, or fewer than 200 or more than 6,000 detected genes. After log-normalization and scaling, the top 3,000 highly variable genes were retained. To account for dataset-specific technical variation across the three scRNA-seq cohorts, batch effects were corrected using Harmony integration. The first 30 principal components were then used for Uniform Manifold Approximation and Projection (UMAP) embedding and Louvain clustering at a resolution of 1.

Clusters were annotated into 21 cell types based on original labels, established bone marrow hematopoietic markers, DotPlot visualization, and module scoring. Marker genes for each cell type were identified using FindAllMarkers with the following thresholds: P < 0.05, |log2FC| > 0.5, and min.pct > 0.25.

Spatial transcriptomics data from GSE279576 sample BM2 were deconvolved using Robust Cell Type Decomposition (RCTD) in doublet mode, with the integrated scRNA-seq dataset serving as the reference. After log-normalization and scaling of the top 10,000 variable genes, UMAP analysis was performed using the first 30 principal components. Pathway module scores were calculated using AddModuleScore. Spatial autocorrelation was assessed using global Moran’s I and the local Getis–Ord Gi* statistic, with neighborhoods defined by the 10th percentile of interspot distance and weighted by inverse distance. Gene–cell type co-localization was evaluated using Spearman correlation, and cell–cell proximity was quantified using an inverse-distance-weighted co-localization score.

### 2.2 Acquisition and preprocessing of bulk transcriptomic data

Bone marrow RNA-seq AML datasets with complete follow-up were collected from BeatAML (n = 344) (23), TCGA (n = 118), and GSE106291 (n = 250) (24). Gene symbols were standardized by retaining the primary identifier, removing multi-mapped probes, and excluding genes with missing expression values. Expression matrices were log2-transformed when the median expression value exceeded 100, or when the expression range exceeded 50 with a minimum value greater than 0.

The three datasets were merged based on intersecting gene symbols. Given the heterogeneity among cohorts and platforms, cohort-related technical variation was corrected using the ComBat algorithm implemented in the sva package, with normalize Between Arrays additionally applied to non-TCGA cohorts. The final integrated expression matrix included 712 samples and 14,017 genes and was used for subsequent subtype identification, prognostic modeling, and validation analyses.

### 2.3 Assessment of polyamine metabolism activity and cell subset abundance

Six polyamine metabolism–related gene sets were retrieved from the Molecular Signatures Database (MSigDB): REACTOME_METABOLISM_OF_POLYAMINES, GOBP_POLYAMINE_BIOSYNTHETIC_PROCESS, GOBP_POLYAMINE_METABOLIC_PROCESS, GOBP_POLYAMINE_TRANSPORT, GOBP_REGULATION_OF_POLYAMINE_TRANSMEMBRANE_TRANSPORT, and GOMF_POLYAMINE_TRANSMEMBRANE_TRANSPORTER_ACTIVITY. After merging and deduplication, a curated signature of 76 unique polyamine metabolism-related genes was generated (**Supplementary Table 1**). Polyamine metabolism scores were calculated for individual cells using AddModuleScore, and cells were classified into high- and low-activity groups according to the median score.

Cell type-specific differences in polyamine activity were quantified using Hedges’ g with 95% confidence intervals (CIs). Enrichment of each cell type in the high- versus low-polyamine group was assessed using odds ratios (ORs) with 95% CIs, with significance evaluated by Fisher’s exact test followed by Benjamini–Hochberg (BH) correction. Differential abundance was further assessed using miloR based on K-nearest-neighbor graph neighborhoods, with spatial false discovery rate (FDR) < 0.05 considered significant. Differences in cell-type proportions between the two groups were also calculated to provide a complementary measure of cellular representation (25).

### 2.4 Analysis of cellular stemness and functional pathway divergence

Transcriptomic stemness scores were calculated for individual cells using CytoTRACE2 (v1.0.0) (26) and projected onto UMAP coordinates to visualize their distribution across the single-cell embedding. Functional state gene sets, including proliferation, inflammation, hypoxia, and DNA damage response, were obtained from the CancerSEA database, whereas Hallmark, Kyoto Encyclopedia of Genes and Genomes (KEGG), and Reactome pathway gene sets were retrieved from MSigDB. Corresponding functional state and pathway activity scores were calculated for each cell using AddModuleScore. Normalized heatmaps were generated to summarize functional profiles across annotated cell populations. Differences in stemness scores and pathway activities between the high- and low-polyamine metabolism groups were assessed using the Wilcoxon rank-sum test, with multiple testing correction performed using the Benjamini–Hochberg method. Adjusted P < 0.05 was considered statistically significant.

### 2.5 Inference of cell–cell communication networks

Cell–cell communication networks among annotated cell types in the high- and low-polyamine metabolism groups were inferred using CellChat (v2.1.2) (27) with its curated human ligand–receptor interaction database. Signaling interactions were aggregated at the cell population level, and both the total number and overall strength of inferred interactions were quantified and compared between groups. Signaling pathways and ligand–receptor (L–R) pairs with differential activity were identified, and the top 20 differential L–R pairs were prioritized according to differences in communication probability (**Supplementary Table 2**). Subnetwork changes involving key cell populations were further visualized to characterize group-specific signaling patterns.

### 2.6 AML blast subset analysis and candidate gene prioritization

The AML blast compartment was extracted from the integrated single-cell dataset and subjected to a second, blast-restricted clustering analysis using Seurat with a Louvain resolution of 0.2 to resolve transcriptional heterogeneity within the malignant blast population. Subcluster marker genes were identified using FindAllMarkers with the following thresholds: adjusted P < 0.05, |log2FC| > 0.5, and min.pct > 0.25 (**Supplementary Table 3**). Polyamine metabolism, functional state, and pathway activity scores were calculated for individual blast cells and summarized across subclusters. Spearman correlation analysis was used to assess associations between the polyamine metabolism score and scores for autophagy, proliferation, MAPK/RAS signaling, monocytic/myeloid differentiation, and DNA damage response, with P < 0.001 considered significant.

Within AML blasts, Spearman correlation analysis was performed between each gene and the polyamine metabolism score to identify positively correlated genes (rho > 0.1, adjusted P < 0.05). In parallel, genes overexpressed in AML blasts compared with all other cell types were identified using FindMarkers (adjusted P < 0.05, log2FC > 0.1). The intersection of these two gene sets was defined as the candidate prognostic gene pool (**Supplementary Table 4**).

### 2.7 Consensus clustering and molecular characterization of bulk transcriptomic profiles

Univariate Cox regression analysis was performed for candidate genes present in the bulk expression matrix, and genes with P < 0.01 were retained for unsupervised clustering (**Supplementary Table 5**). Consensus clustering was performed using ConsensusClusterPlus (v1.68.0) with 80% subsampling, partitioning around medoids, Euclidean distance, and 50 iterations. Candidate cluster numbers from k = 2 to k = 9 were evaluated, and the optimal k was determined based on the consensus matrix and cumulative distribution function curves.

Pathway activity scores for Hallmark, KEGG, Reactome, and WikiPathways gene sets were calculated for each sample. Somatic mutation data were processed using maftools (v2.20.0), and differences in mutation frequencies between clusters were assessed using Fisher’s exact test.

### 2.8 Multi-algorithm prognostic model construction and functional annotation

Ten machine-learning algorithms—random survival forest (RSF), LASSO-Cox, Survival-SVM, SuperPC, ridge regression, elastic net, plsRcox, CoxBoost, stepwise Cox, and gradient boosting machine with Cox distribution (GBM-Cox)—were applied to construct prognostic models. Model performance was assessed using the concordance index (C-index), with GSE106291 serving as the training cohort and BeatAML and TCGA-AML as independent validation cohorts. The optimal model was selected based on overall C-index performance. Samples were dichotomized into high- and low-risk groups using the optimal risk score cutoff. Survival differences were evaluated using Kaplan–Meier curves and the log-rank test. Time-dependent receiver operating characteristic (ROC) curves and area under the curve (AUC) values at 1, 3, and 5 years were calculated using the timeROC package. Risk scores were projected onto single-cell UMAP visualizations.

Gene set enrichment analysis (GSEA) was performed for genes positively and negatively correlated with the risk score, using Gene Ontology (GO) Biological Process, KEGG, Reactome, and Hallmark gene sets as references. Parameters included a gene set size range of 10–500 and 1,000 permutations. Significance thresholds were set at adjusted P < 0.05 and |NES| > 1.

### 2.9 Immune microenvironment profiling and immunotherapy response prediction

Immune cell infiltration abundance and immune signature scores were estimated for bulk samples using the IOBR package (v0.99.8) (28), integrating seven algorithms: MCPcounter, EPIC, CIBERSORT, immunophenoscore (IPS), quanTIseq, ESTIMATE, and TIMER. Tumor Immune Dysfunction and Exclusion (TIDE) scores were calculated together with the T-cell-inflamed signature (TIS), interferon-gamma (IFNG), innate anti-PD-1 resistance (IPRES), M2 macrophage, myeloid-derived suppressor cell (MDSC), and IPS scores. Group comparisons were performed using the Wilcoxon rank-sum test, and differences in predicted immunotherapy responder proportions were assessed using Fisher’s exact test.

### 2.10 Drug sensitivity prediction and gene dependency analysis

Drug sensitivity, represented by the half-maximal inhibitory concentration (IC50), was predicted using pRRophetic (v0.5) (29) based on GDSC2 expression and drug response data, with empirical Bayes batch correction applied and the 20% least variable genes excluded. Differences in predicted IC50 values between risk groups were assessed using the t-test, and associations between predicted IC50 values and risk scores were evaluated using Spearman correlation, with Benjamini–Hochberg (BH) adjustment applied to P values.

Key contributing genes were identified through SHapley Additive exPlanations (SHAP) analysis of the random survival forest model using fastshap (v0.1.1) (30). The top 10 genes ranked by mean absolute SHAP value were retained. Gene essentiality was evaluated using CERES scores from DepMap (The Cancer Dependency Map Project, https://depmap.org/portal/), where lower scores indicate greater dependency. Pearson correlation analysis was used to link CERES scores with functional state and metabolic pathway activity scores, followed by BH correction.

### 2.11 Regulatory network analysis of key genes

Human gene–chemical interaction data were obtained from the Comparative Toxicogenomics Database (CTD, https://ctdbase.org/) to extract chemical entries associated with key genes and construct a bipartite gene–chemical network (**Supplementary Table 6**). High-throughput drug perturbation transcriptomic datasets were integrated to identify key genes significantly altered after drug treatment and to infer compound-specific up- or downregulatory effects (31).

Genome-wide knockout transcriptomic data were retrieved from the GPSA database (https://www.gpsadb.com/). Upstream regulators, defined as genes whose knockout significantly altered the expression of key genes, and downstream targets, defined as genes significantly altered after knockout of key genes, were identified using adjusted P < 0.05 and |log2FC| > 0.5. High-confidence regulatory edges were retained to construct upstream, downstream, and integrated transcriptional regulatory networks.

All networks were visualized using the igraph (v2.2.1) and ggraph (v2.2.2) packages in R. Node size reflected connectivity, edge thickness denoted regulatory strength, and edge color distinguished activation from repression.

### 2.12 Quantitative real-time PCR analysis

Total RNA was extracted from bone marrow samples collected from 12 AML patients and 12 healthy controls using the RNAsimple Total RNA Kit (TIANGEN). cDNA was synthesized from purified RNA using a reverse transcription kit on an Eppendorf Nexus PCR system according to the manufacturer’s instructions. Quantitative real-time PCR was performed using TOROIVD 5G qPCR Premix with UNG on a QuantStudio 5 Real-Time PCR System. β-actin was used as the internal control. Relative mRNA expression levels of CCT6A were calculated using the 2−ΔΔCt method and are presented as mean ± standard error of the mean (SEM). Each reaction was performed in triplicate. Written informed consent was obtained from all participants, and the study was approved by the Ethics Committee of Ruian People’s Hospital (Approval No. YJ2026092). Primer sequences are listed in **Supplementary Table 7**.

### 2.13 Cell culture and small interfering RNA transfection

MOLM-13 cells were purchased from Sunncell and maintained in RPMI-1640 medium supplemented with 10% fetal bovine serum and 1% penicillin–streptomycin at 37 °C in a humidified incubator with 5% CO_2_. Log-phase cells were used for transfection experiments. For CCT6A knockdown, MOLM-13 cells were transfected with CCT6A-specific siRNAs or negative control siRNA using Lipofectamine 3000 reagent in Opti-MEM medium. The final siRNA concentration was 50 nM. After 4–6 h of transfection, the medium was replaced with fresh complete culture medium. Cells were collected 48–72 h later for qPCR and functional assays. Knockdown efficiency was examined by reverse-transcription qPCR (RT-qPCR) using SYBR Green Premix Pro Taq HS qPCR Kit on a LightCycler 96 Real-Time PCR System. Relative CCT6A expression was normalized to β-actin and calculated using the 2−ΔΔCt method, with the negative control siRNA group used as the reference.

### 2.14 Cell viability and spermidine rescue assay

Cell viability was assessed using the Cell Counting Kit-8 (CCK-8) assay. After transfection, MOLM-13 cells were seeded in 96-well plates at 2 × 10^4^ cells per well. At 0, 12, 24, 36, and 48 h, 10 μl of CCK-8 reagent was added, followed by incubation at 37 °C for 30 min, and absorbance was measured at 450 nm. For rescue experiments, CCT6A-silenced cells were treated with 100 μM spermidine and analyzed using the same procedure. Each experiment was independently repeated three times.

### 2.15 Flow cytometric analysis of apoptosis

Apoptosis was evaluated using the fluorescein isothiocyanate (FITC) Annexin V Apoptosis Detection Kit I. After transfection, MOLM-13 cells were harvested, washed twice with cold phosphate-buffered saline (PBS), and resuspended in 1× binding buffer at a concentration of 1 × 10^6^ cells/ml. Then, 100 μl of cell suspension was incubated with 5 μl of FITC Annexin V and 5 μl of propidium iodide (PI) for 15 min at room temperature in the dark. After staining, 400 μl of binding buffer was added, and samples were analyzed within 1 h using a CytoFLEX S flow cytometer. At least 10,000 events were acquired for each sample. The percentages of early and late apoptotic cells were analyzed using FlowJo software, and total apoptosis was defined as the sum of Annexin V-positive/PI-negative and Annexin V-positive/PI-positive cells.

### 2.16 Western blotting

MOLM-13 cells were lysed in radioimmunoprecipitation assay (RIPA) buffer containing protease and phosphatase inhibitors, and protein concentrations were determined by bicinchoninic acid (BCA) assay. Equal amounts of protein (30 μg) were separated by sodium dodecyl sulfate-polyacrylamide gel electrophoresis (SDS-PAGE) and transferred to polyvinylidene difluoride (PVDF) membranes. After blocking, membranes were incubated overnight at 4 °C with antibodies against PCNA, Bcl-2, Bax, and β-actin, followed by horseradish peroxidase (HRP)-conjugated secondary antibodies. Protein bands were visualized by enhanced chemiluminescence (ECL), quantified using ImageJ, and normalized to β-actin. Experiments were independently repeated three times.

### 2.17 Measurement of intracellular polyamines and ODC1 activity

Intracellular putrescine, spermidine, spermine, and ODC1 activity were measured in MOLM-13 cells using commercial enzyme-linked immunosorbent assay (ELISA) kits according to the manufacturers’ instructions. Cells were washed with ice-cold PBS, lysed by sonication, and centrifuged to collect supernatants. Polyamines were quantified by competitive ELISA, whereas ODC1 activity was measured by sandwich ELISA. Absorbance was measured at 450 nm, and concentrations or activity were calculated from standard curves with correction for sample dilution.

### 2.18 Statistical analysis

All statistical analyses were performed in R (v4.4.1). Continuous variables were compared using the Wilcoxon rank-sum test or unpaired Student’s t-test, as appropriate. Correlations were assessed using Spearman’s rank correlation or Pearson’s correlation, depending on data distribution and analysis context. Categorical variables were compared using Fisher’s exact test. Survival differences were evaluated using Kaplan–Meier curves and the log-rank test. Multiple testing correction was performed using the Benjamini–Hochberg procedure, and adjusted P < 0.05 was considered statistically significant.

## 3 Results

### 3.1 Single-cell transcriptomic atlas and cellular composition of AML

**Figure 1:**
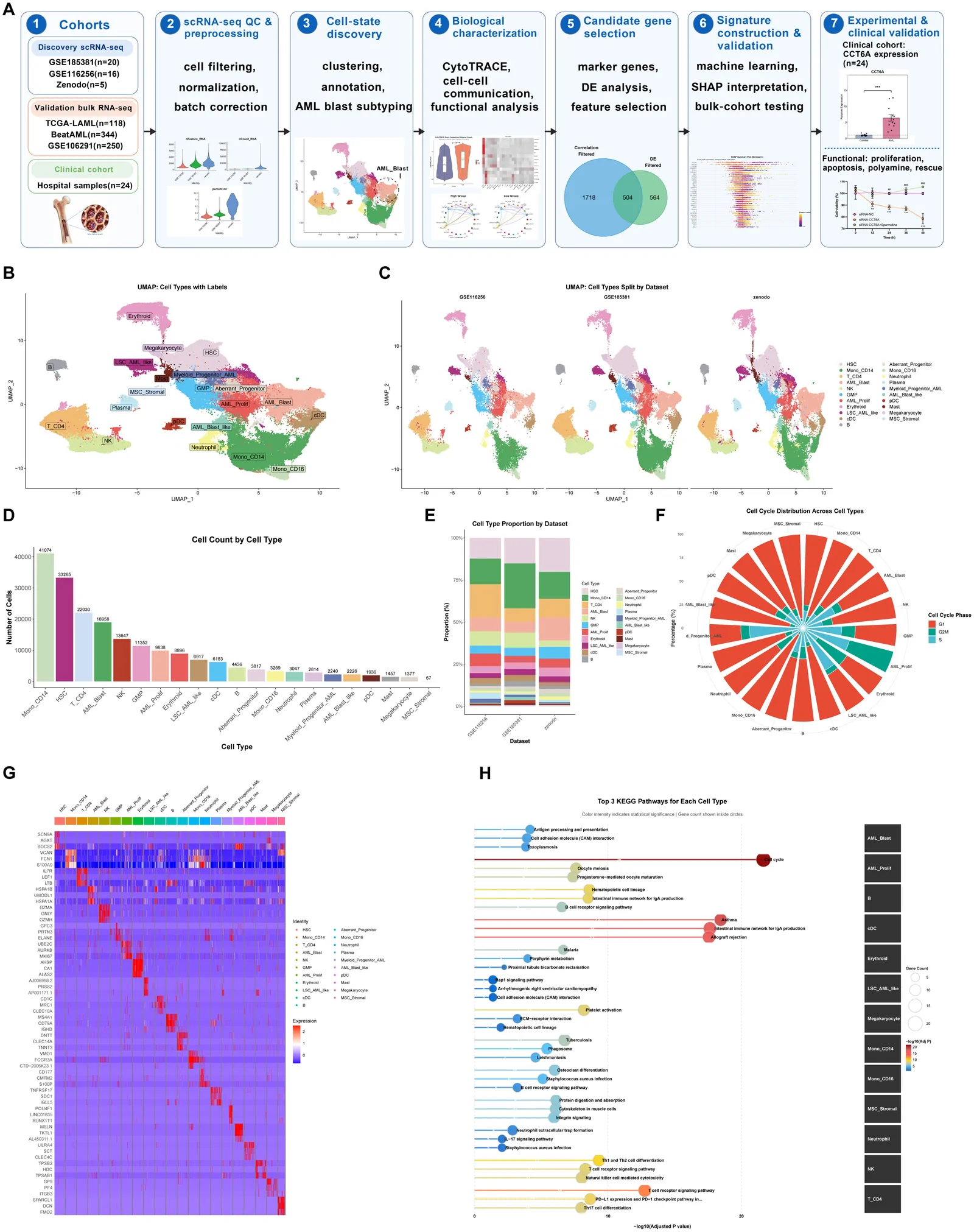
Study workflow and single-cell transcriptomic landscape of AML. **(A)** Overview of the study design and analytical workflow. **(B)** UMAP of integrated single-cell data colored by annotated cell type. **(C)** Split UMAP showing cells from the three scRNA-seq datasets. **(D)** Cell numbers across annotated cell types. **(E)** Cell-type proportions across datasets. **(F)** Cell-cycle phase distribution across cell types. **(G)** Heatmap of representative cell type-specific marker genes. **(H)** KEGG pathway enrichment across representative cell populations.

The overall study design is summarized in **Figure 1A**. We integrated three independent scRNA-seq datasets (GSE116256, GSE185381, and Zenodo), comprising 41 AML samples and 198,846 cells. UMAP analysis resolved 21 annotated cell populations and revealed substantial cellular heterogeneity within the AML bone marrow microenvironment (**Figure 1B**). Following integration, cells from the three datasets showed broadly overlapping distributions across major cell populations, consistent with a substantial reduction in dataset-specific effects (**Figure 1C**). Cell abundance and composition varied across populations and datasets, with CD14+ monocytes, HSCs, CD4+ T cells, and AML blasts representing major cellular compartments (**Figures 1D, E**).

Cell-cycle composition differed across cell types, with proliferative AML cells showing the greatest enrichment of actively cycling states (**Figure 1F**). Cell type-specific marker expression further supported the assigned cellular identities (**Figure 1G**). KEGG enrichment analysis revealed distinct functional programs across populations, including prominent cell-cycle activity in proliferative AML cells and immune-related pathways in lymphoid and myeloid immune populations (**Figure 1H**). Together, these analyses established a cellular and functional reference atlas for subsequent characterization of polyamine metabolism.

### 3.2 Polyamine metabolism activity and differential cellular abundance in AML

**Figure 2:**
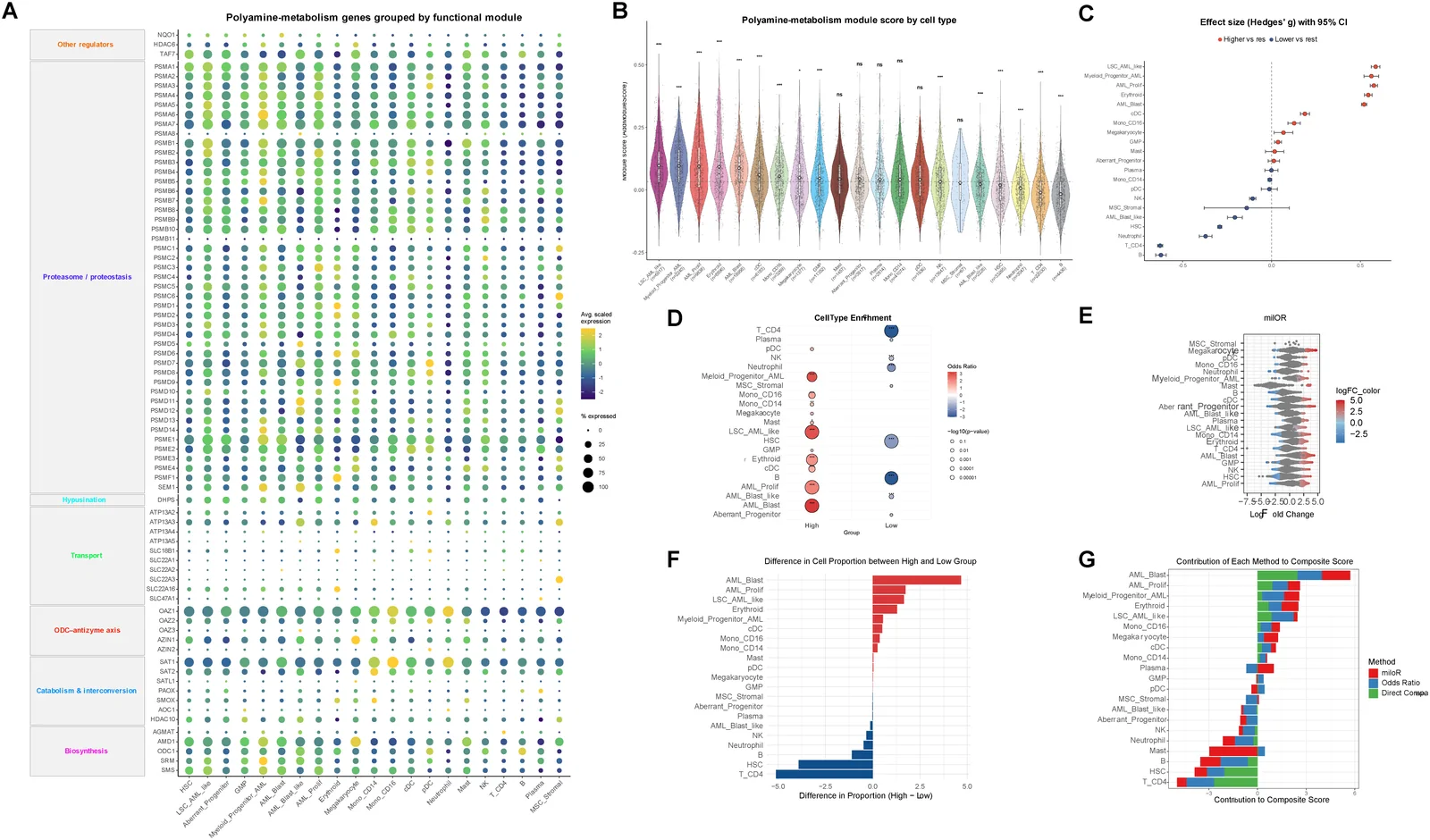
Polyamine metabolism activity and differential cellular abundance in AML. **(A)** Dot plot of 76 polyamine metabolism-related genes across 21 cell types, grouped by functional module. **(B)** Violin plots of polyamine metabolism scores; significance was assessed by Welch’s t-test with BH adjustment. **(C)** Hedges’ g effect sizes with 95% CIs for each cell type versus all remaining cells. **(D)** Cell-type enrichment in high- versus low-polyamine groups based on odds ratios and BH-adjusted significance. **(E)** miloR differential abundance analysis between high- and low-polyamine groups. **(F)** Differences in cell-type proportions between the two groups. **(G)** Composite ranking integrating proportion differences, odds ratios, and miloR results. *P < 0.05, **P < 0.01, ***P < 0.001; ns, not significant.

To characterize polyamine metabolism across the AML bone marrow microenvironment, we curated a 76-gene signature and grouped its components into functional modules, including biosynthesis, catabolism/interconversion, the ornithine decarboxylase (ODC)–antizyme axis, transport, hypusination, and proteostasis-related processes, with representative genes including ODC1, SRM, SMS, SAT1, OAZ1, and EIF5A (**Figure 2A**). Polyamine metabolism scores varied markedly across cell types (**Figure 2B**). Effect-size analysis showed higher activity in leukemia stem cell (LSC)-like, proliferative AML, AML progenitor, and AML blast populations (Hedges’ g = 0.586, 0.576, 0.562, and 0.521, respectively), whereas CD4+ T and B cells showed lower activity (g = −0.627 and −0.622; BH-adjusted P < 2.2 × 10^−16^; **Figure 2C**; **Supplementary Table 8**).

Cells were subsequently stratified into high- and low-polyamine groups. AML blasts showed the strongest enrichment in the high-polyamine group (OR = 3.24, 95% CI 3.13–3.35), followed by LSC-like cells and AML progenitors (both OR = 2.91) and proliferative AML cells (OR = 2.15), whereas CD4+ T and B cells were preferentially enriched in the low-polyamine group (both OR ≈ 0.33; **Figure 2D**; **Supplementary Table 8**). miloR identified 3,857 significantly differentially abundant neighborhoods, including 2,743 enriched in the high-polyamine group and 1,114 in the low-polyamine group, with malignant/progenitor neighborhoods showing predominantly positive shifts and mature lymphoid populations showing the opposite pattern (**Figure 2E**; **Supplementary Table 9**). Cell-proportion comparisons showed a concordant distribution (**Figure 2F**). Integration of the three abundance measures ranked AML blasts highest, followed by proliferative and progenitor-like AML populations (**Figure 2G**). These findings link elevated polyamine activity to primitive and proliferative AML cell states.

### 3.3 Stemness and functional alterations associated with high polyamine metabolism

**Figure 3:**
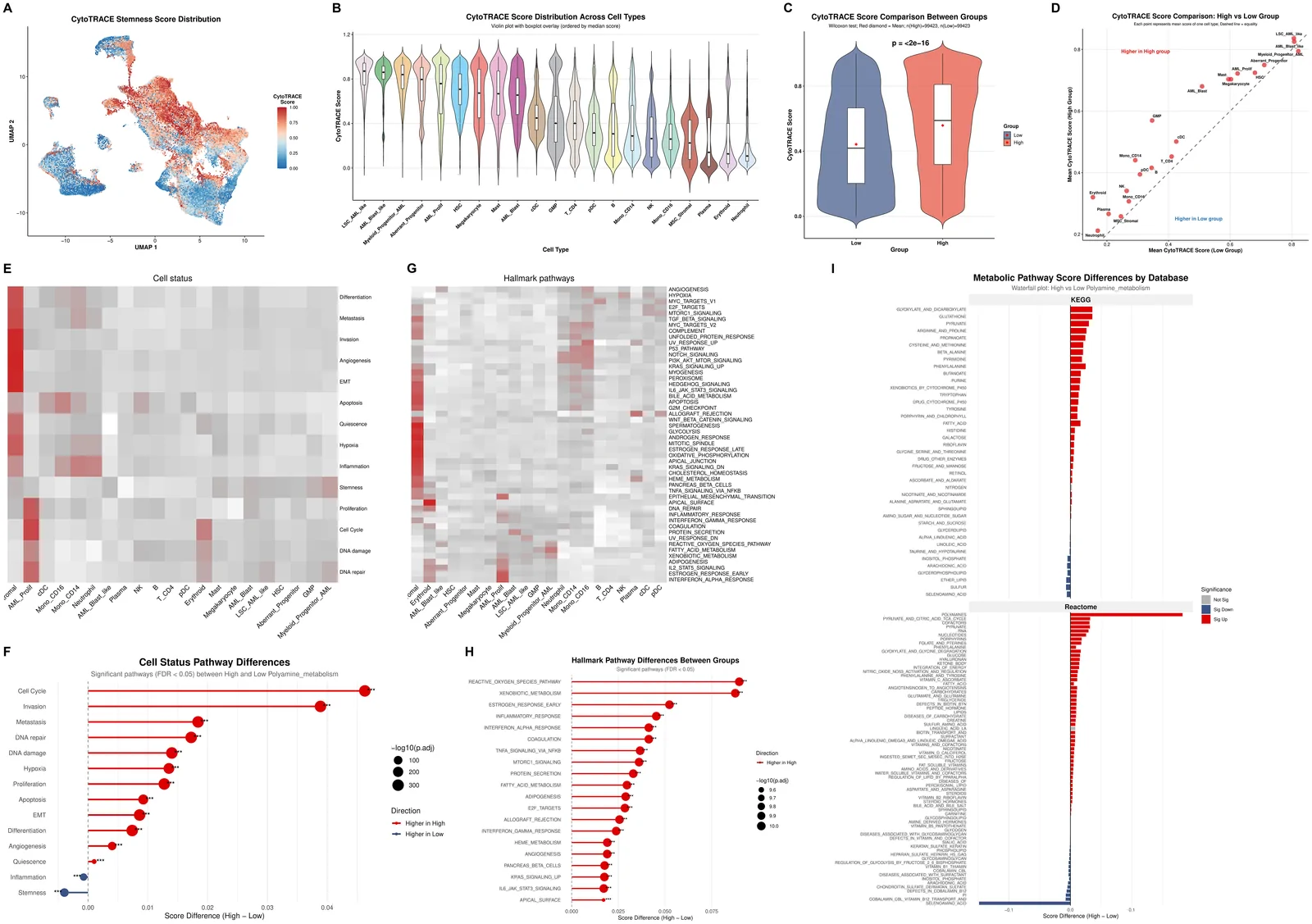
Stemness and functional pathway differences associated with polyamine metabolism. **(A)** UMAP projection colored by CytoTRACE stemness score. **(B)** Violin plots showing CytoTRACE scores across annotated cell types. **(C)** Violin plot comparing CytoTRACE scores between the high- and low-polyamine metabolism groups. **(D)** Scatter plot comparing mean CytoTRACE scores between the high- and low-polyamine metabolism groups across cell types. **(E)** Heatmap showing CancerSEA cell-state scores across annotated cell types. **(F)** Bar plot showing differences in CancerSEA cell-state scores between the high- and low-polyamine metabolism groups. **(G)** Heatmap showing Hallmark pathway scores across annotated cell types. **(H)** Bar plot showing Hallmark pathways with significant score differences between groups (FDR < 0.05). **(I)** Waterfall plot showing KEGG and Reactome metabolic pathway score differences between the high- and low-polyamine metabolism groups. ***P < 0.001.

We examined stemness characteristics across the AML bone marrow microenvironment using CytoTRACE. UMAP projection revealed a continuous gradient of developmental potential across cell populations (**Figure 3A**). CytoTRACE scores were highest in LSC-like cells (median = 0.871), AML blast-like cells (0.861), and AML progenitors (0.840), whereas mature neutrophil, erythroid, and plasma-cell populations showed substantially lower scores (medians = 0.108, 0.127, and 0.141, respectively; **Figure 3B**; **Supplementary Table 10**). High-polyamine cells showed higher CytoTRACE scores than low-polyamine cells (mean 0.558 vs. 0.442; Wilcoxon P < 2 × 10^−16^; **Figure 3C**). At the cell-type level, mean CytoTRACE scores were higher in the high-polyamine group in 20 of 21 annotated cell types, including AML blasts (0.680 vs. 0.509) and proliferative AML cells (0.722 vs. 0.624), whereas AML progenitors showed the opposite pattern (0.795 vs. 0.821; **Figure 3D**; **Supplementary Table 11**).

Functional state analysis using CancerSEA gene sets revealed marked heterogeneity in proliferation, inflammation, hypoxia, and related programs across cell populations (**Figure 3E**). Most significantly altered functional states showed higher activity in the high-polyamine group after BH correction (**Figure 3F**). Hallmark, KEGG, and Reactome analyses similarly revealed broad pathway differences between the two groups, with multiple pathways showing increased activity in high-polyamine cells (**Figures 3G–I**). Together, these findings associate elevated polyamine metabolism with increased stemness and broader functional activation in AML.

### 3.4 Cell–cell communication network differences between high- and low-polyamine metabolism groups

**Figure 4:**
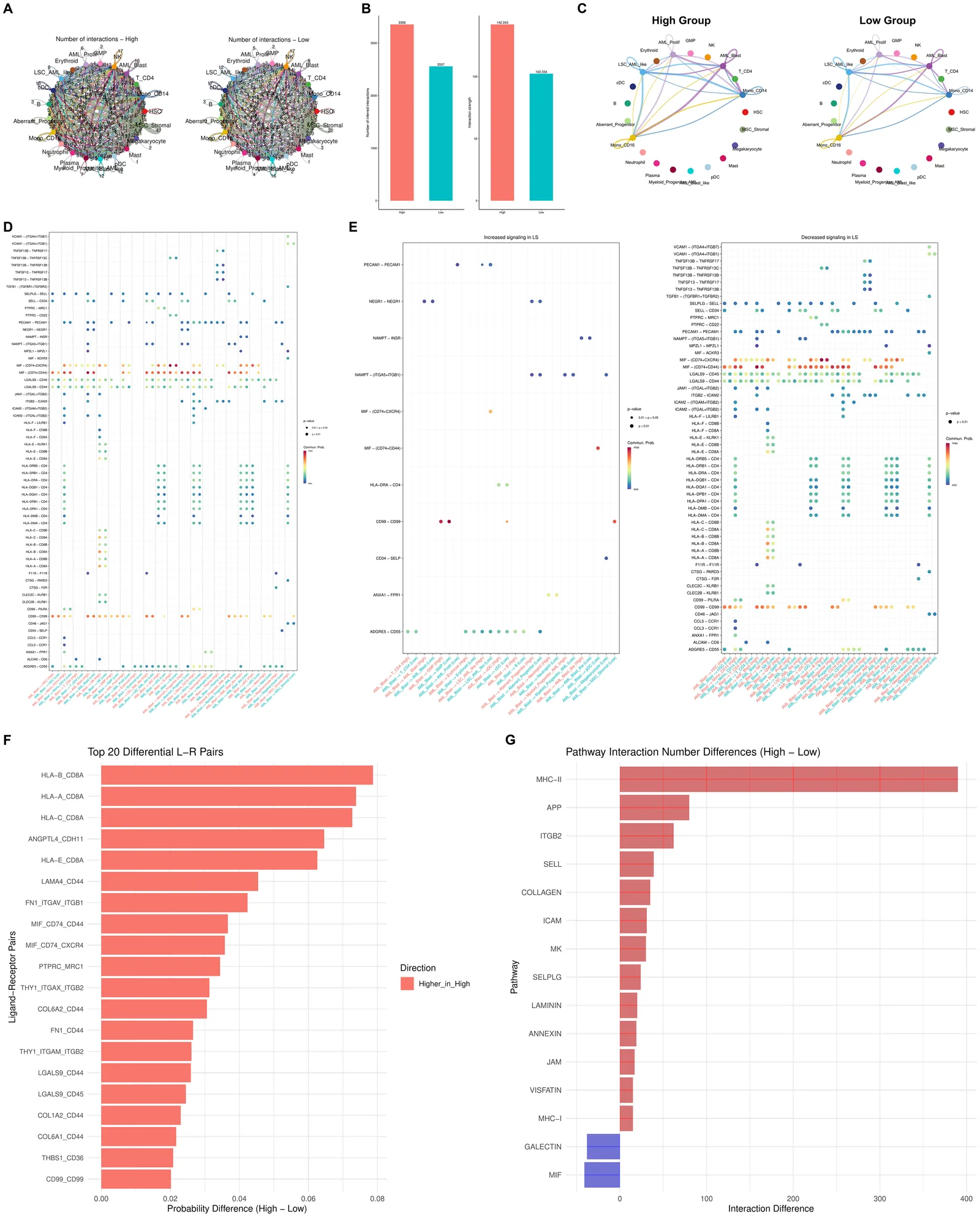
Cell–cell communication differences between high- and low-polyamine metabolism groups. **(A)** Circle plots showing inferred cell–cell communication networks in the high- and low-polyamine metabolism groups. **(B)** Bar plots comparing the total number of interactions and overall communication strength between groups. **(C)** Subnetwork visualization of selected signaling interactions among major cell populations in each group. **(D)** Bubble plot showing signaling pathways with differential activity between groups. **(E)** Dot plots showing AML blast-derived ligand–receptor pairs with increased or decreased communication probabilities between groups. **(F)** Bar plot ranking the top 20 differential ligand–receptor pairs by communication probability difference; all listed pairs were elevated in the high-polyamine metabolism group. **(G)** Bar plot showing differences in signaling pathway interaction numbers between the high- and low-polyamine metabolism groups.

CellChat analysis revealed a denser inferred intercellular communication network in the high-polyamine metabolism group, with increased interaction numbers and overall communication strength compared with the low-polyamine group (**Figures 4A–C**). Differential pathway analysis further identified multiple signaling programs with altered communication activity between groups (**Figure 4D**). AML blast-derived ligand–receptor interactions showed both increased and decreased communication probabilities, indicating bidirectional remodeling rather than uniform activation (**Figure 4E**).

The top 20 differential interactions were all elevated in the high-polyamine group, with HLA class I-related pairs, including HLA-B_CD8A, HLA-A_CD8A, and HLA-C_CD8A, ranking prominently (**Figure 4F**). At the pathway level, MHC-II, APP, and ITGB2 signaling showed the largest increases in interaction number, whereas relatively few pathways were increased in the low-polyamine group (**Figure 4G**). Together, these findings indicate that elevated polyamine metabolism is associated with broader and altered inferred intercellular communication in AML.

### 3.5 Heterogeneity and functional modules of AML blasts

**Figure 5:**
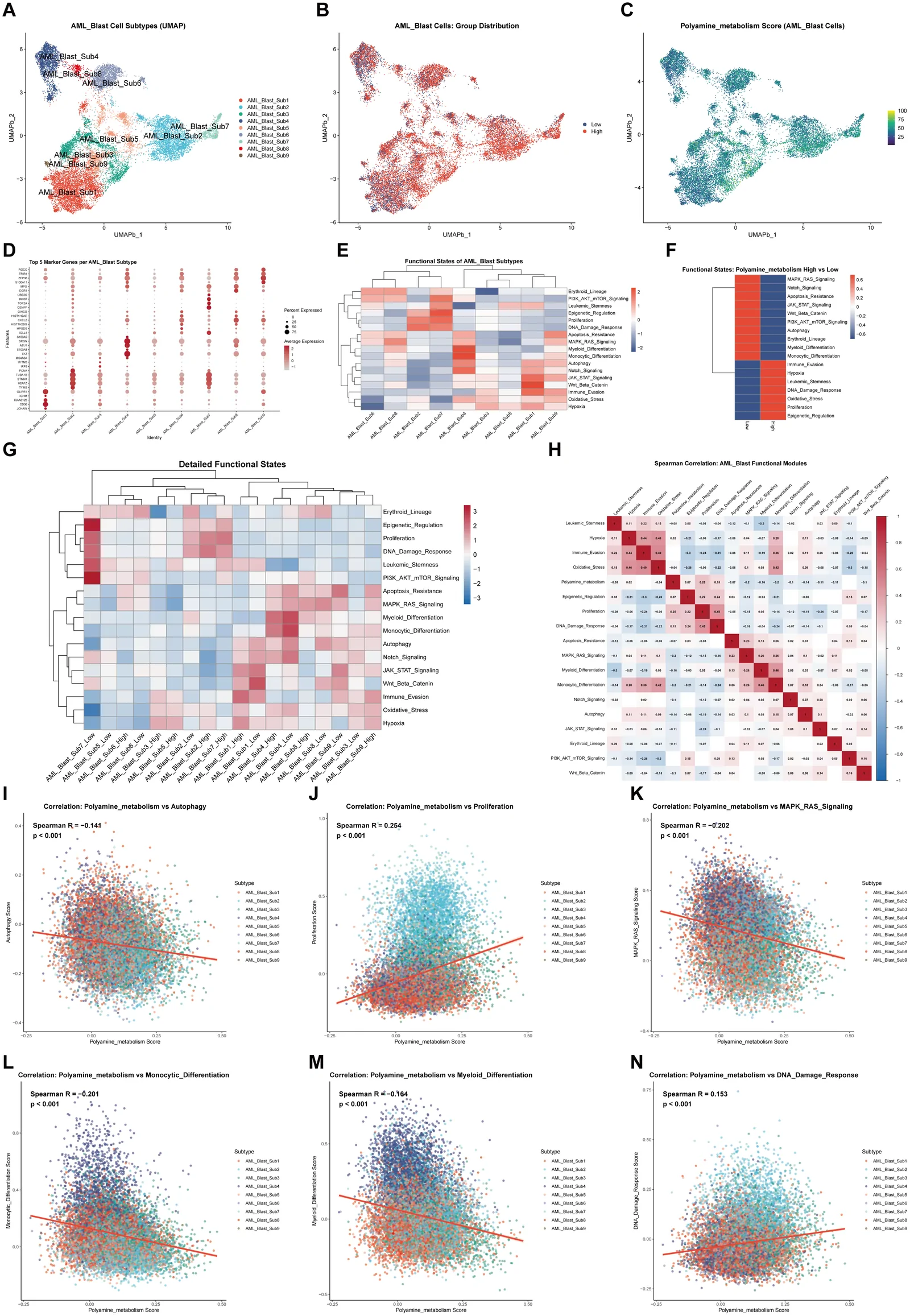
AML blast subpopulations and polyamine metabolism-associated functional states. **(A)** UMAP visualization of nine re-clustered AML blast subpopulations (Sub1–Sub9). **(B)** UMAP visualization of AML blast cells colored by high- or low-polyamine metabolism group. **(C)** UMAP visualization of AML blast cells colored by polyamine metabolism score. **(D)** Dot plot showing subpopulation-specific marker gene expression. **(E)** Heatmap showing functional module scores across AML blast subpopulations. **(F)** Heatmap comparing functional module scores between the high- and low-polyamine metabolism groups. **(G)** Heatmap showing refined functional state scores with hierarchical clustering. **(H)** Spearman correlation matrix of functional modules in AML blast cells. **(I–N)** Scatter plots showing Spearman correlations between the polyamine metabolism score and autophagy **(I)**, proliferation **(J)**, MAPK/RAS signaling **(K)**, monocytic differentiation **(L)**, myeloid differentiation **(M)**, and DNA damage response **(N)** scores.

Because AML blasts showed the strongest association with elevated polyamine metabolism, we next isolated the AML blast compartment and performed a second round of clustering within this population to resolve transcriptional heterogeneity among malignant blasts. This blast-restricted analysis identified nine AML blast subpopulations (Sub1–Sub9) with distinct transcriptional profiles (**Figure 5A**). High- and low-polyamine cells were unevenly distributed across these subpopulations (**Figure 5B**), while polyamine metabolism scores varied continuously across blast cells, indicating a spectrum of metabolic states rather than discrete categories (**Figure 5C**). Subpopulation-specific marker expression further supported the transcriptional heterogeneity of these blast states (**Figure 5D**).

Functional module scoring revealed distinct activation patterns across the nine subpopulations and between high- and low-polyamine groups (**Figures 5E–G**). Inter-module correlations further indicated coordinated functional programs (**Figure 5H**). Polyamine metabolism was negatively correlated with autophagy (R = −0.141, P < 0.001), MAPK/RAS signaling (R = −0.202, P < 0.001), monocytic differentiation (R = −0.201, P < 0.001), and myeloid differentiation (R = −0.164, P < 0.001), but positively correlated with proliferation (R = 0.254, P < 0.001) and DNA damage response (R = 0.153, P < 0.001) (**Figures 5I–N**). These findings associate elevated polyamine metabolism with a proliferative, DNA damage-responsive, and less differentiated AML blast state.

### 3.6 Polyamine-associated candidate genes and bulk transcriptomic characterization

**Figure 6:**
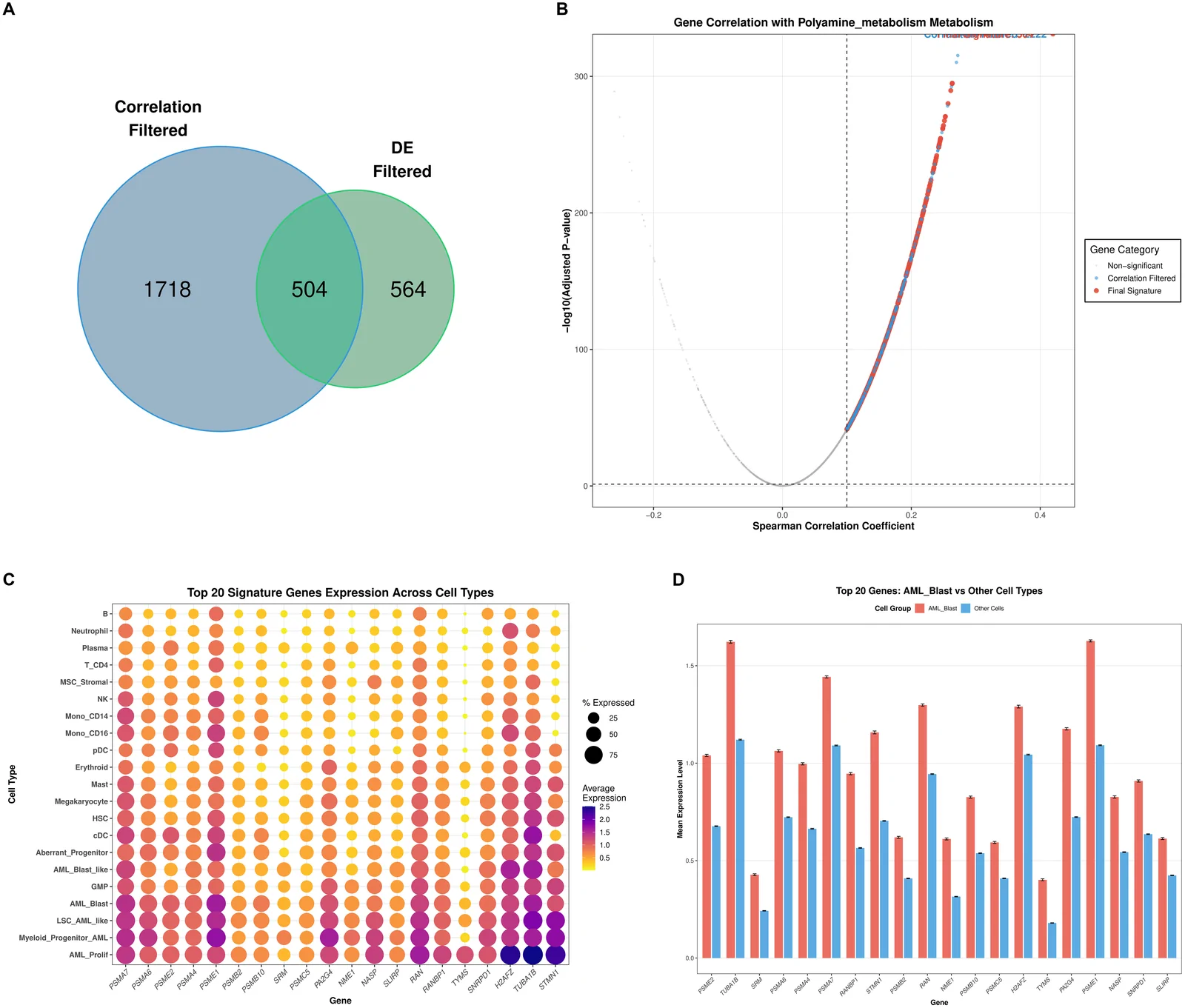
Identification of polyamine metabolism-associated candidate genes in AML blasts. **(A)** Venn diagram showing the intersection between genes positively correlated with the polyamine metabolism score in AML blasts (Spearman rho > 0.1, adjusted P < 0.05) and genes upregulated in AML blasts compared with all other cell types (adjusted P < 0.05, log_2_FC > 0.1), yielding 504 candidate genes. **(B)** Volcano plot showing Spearman correlation coefficients between gene expression and the polyamine metabolism score in AML blasts. Genes are colored according to filtering category, and the 504 intersection genes are highlighted as candidate genes. **(C)** Dot plot showing the average expression and percentage of expressing cells for the top 20 candidate genes across annotated cell types. **(D)** Bar plot comparing the expression levels of the top 20 candidate genes between AML blasts and other cell types, confirming blast-enriched expression.

To connect the single-cell metabolic phenotype with patient-level transcriptional features, we next identified genes associated with both polyamine activity and the AML blast phenotype. Among 2,222 genes positively correlated with polyamine metabolism scores and 1,068 genes upregulated in AML blasts, 504 overlapping candidates were identified (**Figures 6A–D**).

**Figure 7:**
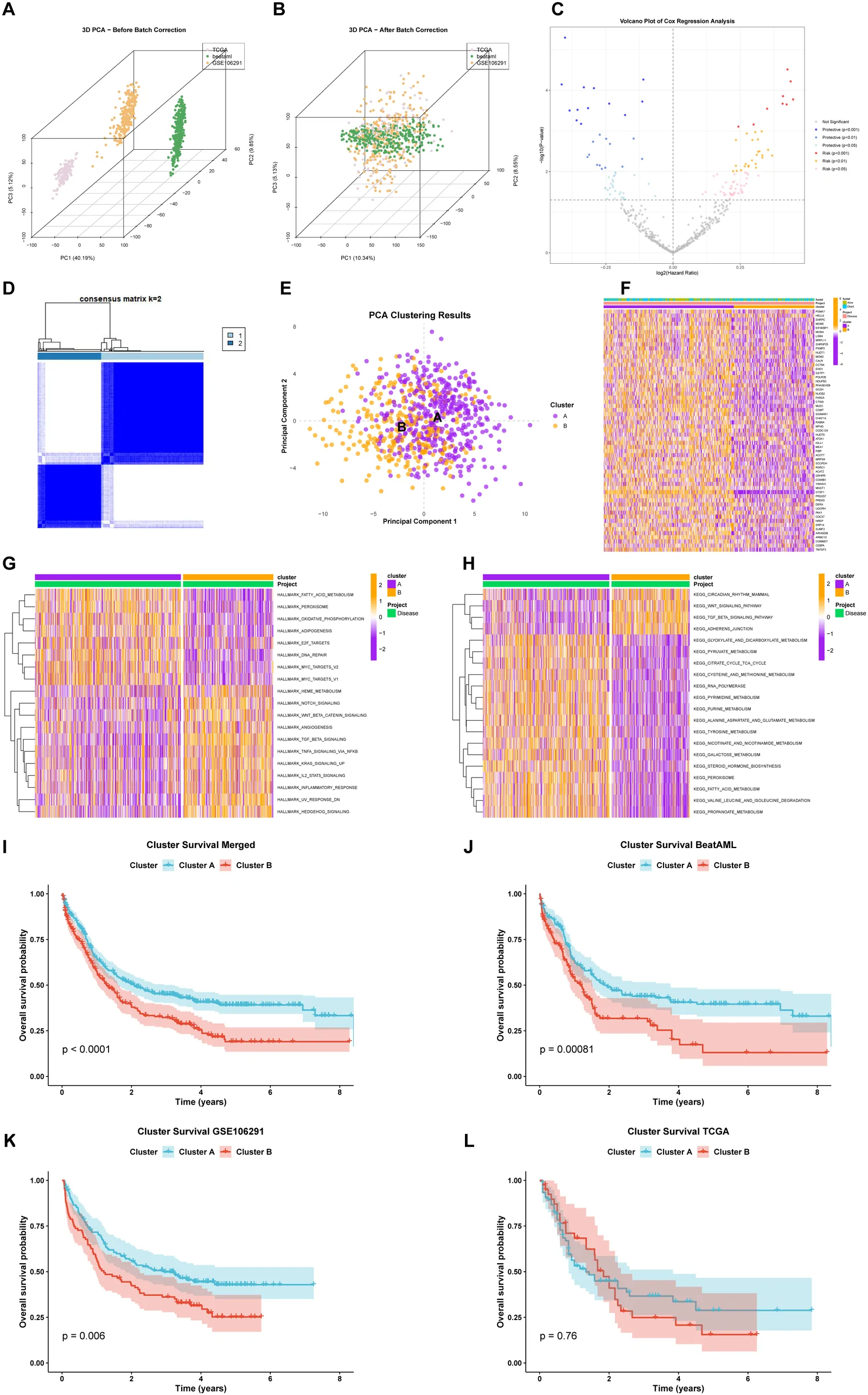
Molecular subtype identification, biological characterization, and survival relevance in AML. **(A, B)** 3D PCA of the three bulk RNA-seq cohorts before and after batch correction. **(C)** Univariate Cox regression screening of survival-associated candidate genes. **(D)** Consensus clustering matrix at k = 2 defining Cluster A and Cluster B. **(E)** PCA showing the distribution of the two molecular subtypes. **(F)** Heatmap of survival-associated genes across the two clusters. **(G, H)** Hallmark and KEGG pathway profiles of Cluster A and Cluster B. **(I–L)** Kaplan–Meier overall survival analyses in the pooled cohort, BeatAML, GSE106291, and TCGA-AML, respectively.

We then evaluated these candidates in the integrated TCGA-AML, BeatAML, and GSE106291 cohorts. Principal component analysis (PCA) showed clear cohort-related separation before correction, with substantially improved overlap after ComBat adjustment (**Figures 7A, B**), indicating a marked reduction in cohort-associated technical variation. Of the 504 candidates, 469 were detected in the integrated dataset, and 59 were associated with overall survival by univariate Cox regression (P < 0.01; **Figure 7C**). Consensus clustering based on these genes identified two molecular subtypes, Cluster A and Cluster B, with distinct transcriptional profiles (**Figures 7D–F**).

Pathway analysis showed that Cluster A was enriched for metabolic and proliferative programs, including oxidative phosphorylation, fatty acid metabolism, MYC/E2F targets, and DNA repair, whereas Cluster B displayed stronger immune-inflammatory, developmental, and stemness-related features (**Figures 7G, H**). Cluster B was associated with poorer overall survival in the pooled cohort, BeatAML, and GSE106291 (all P < 0.01; **Figures 7I–K**), whereas no significant survival difference was observed in TCGA-AML (P = 0.76; **Figure 7L**).

In TCGA-AML, the two subtypes also differed in overall mutation burden, AML-associated mutation patterns, cytogenetic risk, immune infiltration, CellStat, and stemness profiles (**Supplementary Figures 1A–G**). Together, these findings extend the single-cell polyamine-associated blast phenotype to patient-level molecular heterogeneity, defining a metabolically proliferative Cluster A and a stem-like, immune-inflamed Cluster B with cohort-dependent prognostic relevance.

### 3.7 Prognostic risk model performance and single-cell mapping

**Figure 8:**
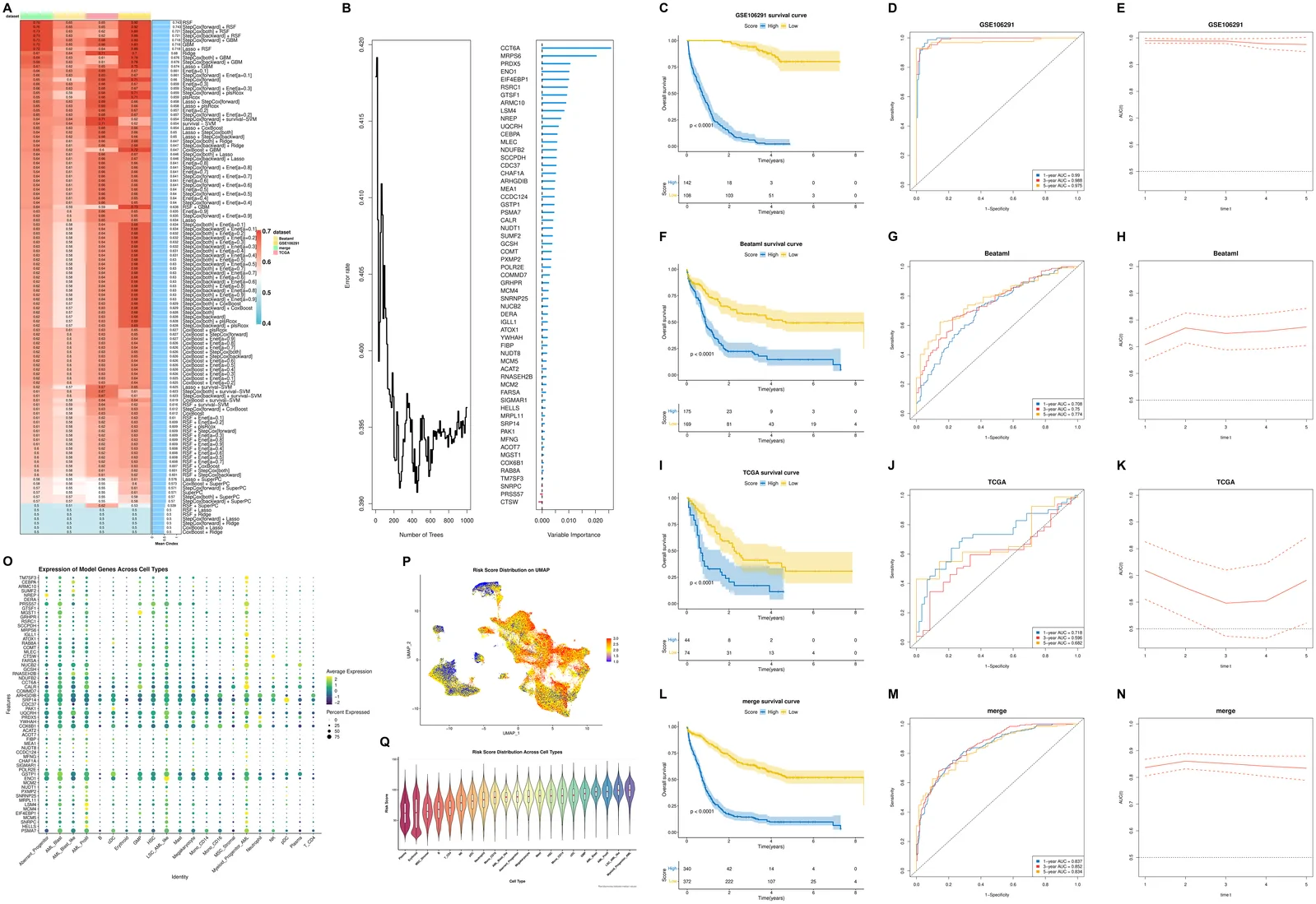
Prognostic model performance and single-cell mapping of risk scores. **(A)** Heatmap showing C-index values for ten machine-learning algorithms and their model combinations across cohorts. **(B)** Feature importance ranking from the Random Survival Forest model. **(C–E)** Kaplan–Meier survival curves, 1-, 3-, and 5-year ROC curves, and time-dependent AUC curves for the GSE106291 cohort. **(F–H)** Kaplan–Meier survival curves, 1-, 3-, and 5-year ROC curves, and time-dependent AUC curves for the BeatAML cohort. **(I–K)** Kaplan–Meier survival curves, 1-, 3-, and 5-year ROC curves, and time-dependent AUC curves for the TCGA-AML cohort. **(L–N)** Kaplan–Meier survival curves, 1-, 3-, and 5-year ROC curves, and time-dependent AUC curves for the merged cohort. **(O)** Dot plot showing model gene expression across annotated cell types. **(P)** UMAP visualization showing the single-cell distribution of risk scores. **(Q)** Violin plot showing risk score distributions across annotated cell types.

Ten machine-learning algorithms, including RSF, LASSO-Cox, Survival-SVM, SuperPC, ridge regression, elastic net, plsRcox, CoxBoost, stepwise Cox, and GBM-Cox, were evaluated using GSE106291 as the training cohort and BeatAML and TCGA-AML as validation cohorts. RSF showed the best overall performance, with a C-index of 0.743 (**Figure 8A**), and the contributions of individual model genes were further evaluated by feature importance analysis (**Figure 8B**). In the training cohort, high-risk patients had significantly shorter overall survival than low-risk patients (log-rank P < 0.001; **Figure 8C**), with 1-, 3-, and 5-year AUCs of 0.990, 0.988, and 0.975, respectively (**Figures 8D, E**).

In BeatAML, the 1-, 3-, and 5-year AUCs were 0.708, 0.750, and 0.774, respectively, with significant survival separation between risk groups (log-rank P < 0.001; **Figures 8F–H**). In TCGA-AML, the corresponding AUCs were 0.718, 0.596, and 0.682, with poorer survival in the high-risk group (log-rank P < 0.001; **Figures 8I–K**). Pooled analysis of the three cohorts yielded AUCs of 0.837, 0.852, and 0.834 at 1, 3, and 5 years, respectively (log-rank P < 0.0001; **Figures 8L–N**). At the single-cell level, model genes showed heterogeneous expression across cell types (**Figure 8O**), and projected risk scores were higher in leukemic blast and progenitor populations than in immune or stromal compartments (**Figures 8P, Q**). These findings support the prognostic robustness of the model and link its transcriptional features predominantly to malignant blast and progenitor states.

### 3.8 Biological characteristics and exploratory therapeutic associations of the risk score

Genes correlated with the risk score showed distinct expression patterns, and GSEA revealed differential pathway enrichment between the two risk groups (**Supplementary Figures 2A–F**). High-risk samples were characterized by stronger inflammatory and neutrophil-related programs, including TNF-α/NF-κB signaling, whereas low-risk samples showed greater enrichment of RNA processing and translation-related pathways.

Immune profiling further indicated a more stromal- and myeloid-enriched microenvironment in the high-risk group, accompanied by increased immune dysfunction and suppressive features, whereas the low-risk group showed relatively greater lymphoid representation and higher immunophenoscore (**Supplementary Figures 3A–K**). Together, these findings suggest distinct immune microenvironmental states associated with the risk model.

In silico drug sensitivity analysis further demonstrated differential predicted drug-sensitivity patterns between the two risk groups. High-risk samples showed higher predicted IC50 values for multiple compounds (FDR < 0.05; **Supplementary Figure 4A**). Representative agents, including Sepantronium bromide, Ulixertinib, AZ6102, and AMG.319, exhibited higher predicted IC50 values in high-risk samples (**Supplementary Figure 4B**), and several compounds showed positive correlations between predicted IC50 values and risk scores (Spearman R = 0.317–0.347; **Supplementary Figures 4C–H**). These findings suggest that the risk score may reflect differences in predicted drug sensitivity among AML patients, although these computational predictions require experimental validation.

### 3.9 Model interpretation and identification of CCT6A as a candidate gene

**Figure 9:**
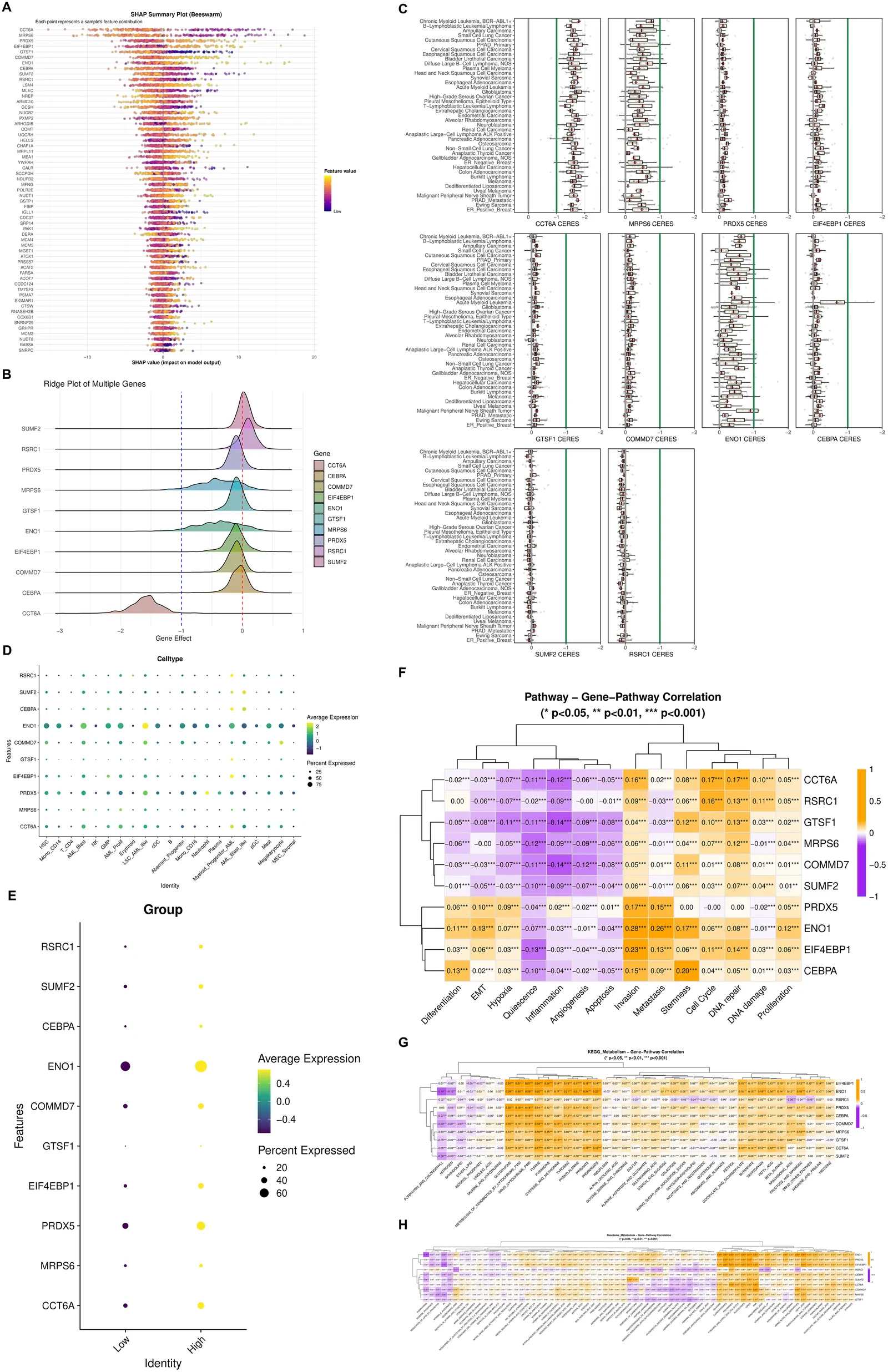
SHAP-based model interpretation, DepMap CERES dependency, and pathway correlations for the RSF model. **(A)** SHAP beeswarm plot showing feature contributions in the RSF model, with genes ranked by mean absolute SHAP value. **(B)** Ridge plot showing DepMap CERES dependency score distributions for the top 10 key genes across cancer types. **(C)** Box plots showing CERES scores of the top 10 key genes across selected cancer types. **(D)** Dot plot showing the expression of the top 10 key genes across annotated cell types. **(E)** Dot plot comparing the expression of the top 10 key genes between the high- and low-risk groups. **(F)** Heatmap showing Pearson correlations between key genes and functional state scores. **(G)** Heatmap showing Pearson correlations between key genes and KEGG metabolic pathway scores. **(H)** Heatmap showing Pearson correlations between key genes and Reactome metabolic pathway scores.

SHAP analysis of the RSF model identified CCT6A, EIF4EBP1, PRDX5, MRPS6, ENO1, COMMD7, CEBPA, GTSF1, SUMF2, and RSRC1 as the top 10 contributing genes (**Figure 9A**). Among these genes, MRPS6, ENO1, and CCT6A showed negative CERES scores in AML cell lines, indicating potential dependency relationships, with CCT6A exhibiting the strongest dependency signal (**Figures 9B, C**).

Single-cell mapping demonstrated heterogeneous expression patterns of the model genes across AML bone marrow cell populations, and several genes, including CCT6A, ENO1, and PRDX5, showed higher expression in high-risk samples (**Figures 9D, E**). Correlation analysis further revealed that model genes were associated with malignant cellular programs, including proliferation, stemness, cell-cycle regulation, DNA repair, and metabolic activity (**Figures 9F–H**). Integrated model contribution, dependency analysis, and association with AML-related biological programs collectively identified CCT6A as the leading candidate gene for subsequent functional validation.

### 3.10 Exploratory analyses of model gene regulation and spatial distribution

Exploratory CTD and perturbation-based analyses further characterized potential gene–chemical associations and regulatory relationships among the model genes (**Supplementary Figure 5**). In addition, spatial transcriptomic analysis using RCTD deconvolution provided complementary spatial context for AML blasts and model gene expression patterns. AML blasts showed heterogeneous spatial localization, and selected model genes, particularly ENO1 and PRDX5, displayed spatial associations with specific cellular populations (**Supplementary Figure 6**). These exploratory analyses provide complementary hypothesis-generating context for the biological relevance of the model genes.

### 3.11 Experimental validation of CCT6A function and polyamine metabolism in AML cells

**Figure 10:**
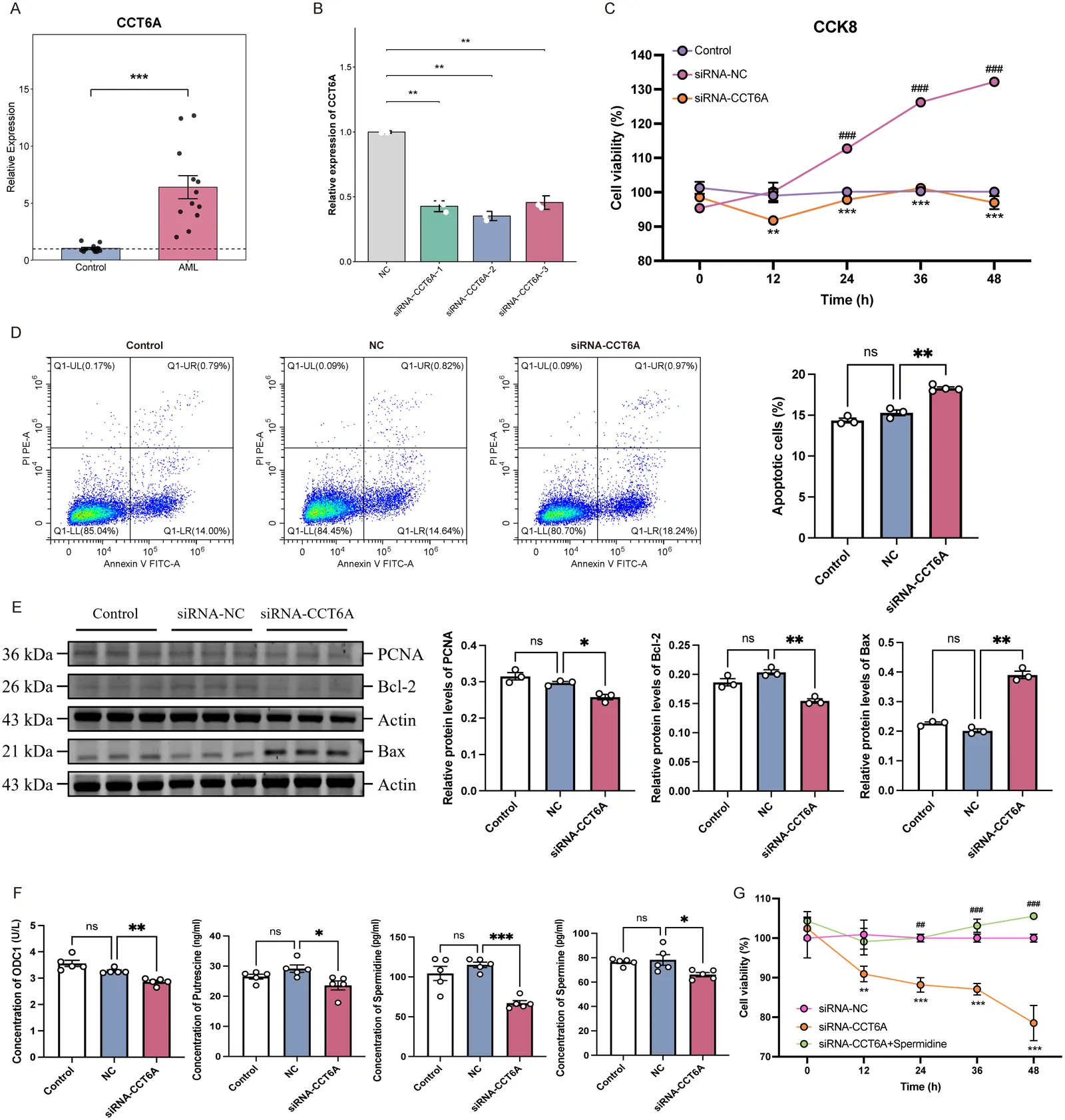
CCT6A supports AML cell viability and polyamine metabolism. **(A)** RT-qPCR analysis of CCT6A expression in healthy controls and patients with AML. **(B)** RT-qPCR validation of three CCT6A-targeting siRNAs. siRNA-CCT6A-2 showed the highest knockdown efficiency and was used for subsequent experiments. **(C)** CCK-8 assay showing reduced cell viability after CCT6A knockdown. Cell viability was normalized to the Control group at each time point (Control = 100%). **(D)** Annexin V-FITC/PI analysis showing increased apoptosis after CCT6A knockdown. **(E)** Western blot analysis showing decreased PCNA and Bcl-2 and increased Bax expression following CCT6A knockdown. **(F)** Measurement of ODC1 activity and intracellular polyamines. CCT6A knockdown reduced ODC1 activity and putrescine, spermine, and spermidine levels, with the most pronounced reduction observed in spermidine. **(G)** CCK-8 rescue assay showing that 100 μM spermidine restored cell viability following CCT6A knockdown. Cell viability was normalized to the siRNA-NC group at each time point (siRNA-NC = 100%). Data are presented as mean ± SEM in **(A)** and mean ± SD in **(B–G)**. ns, not significant. In **(C)**, *P < 0.05, **P < 0.01, and ***P < 0.001 indicate comparisons between siRNA-CCT6A and siRNA-NC; ##P < 0.01 and ###P < 0.001 indicate comparisons between siRNA-NC and Control. In **(G)**, **P < 0.01 and ***P < 0.001 indicate comparisons between siRNA-CCT6A and siRNA-NC; ##P < 0.01 and ###P < 0.001 indicate comparisons between siRNA-CCT6A + spermidine and siRNA-CCT6A.

qPCR analysis showed significantly higher CCT6A expression in AML bone marrow samples than in controls (**Figure 10A**). Three CCT6A-targeting siRNAs efficiently suppressed CCT6A expression in MOLM-13 cells, with siRNA-CCT6A-2 selected for subsequent experiments based on knockdown efficiency (**Figure 10B**). CCT6A silencing significantly reduced cell viability and increased apoptosis compared with the siRNA-NC group (**Figures 10C, D**). Consistently, Western blotting showed decreased PCNA and Bcl-2 expression and increased Bax expression after CCT6A knockdown (**Figure 10E**). CCT6A silencing also reduced ODC1 activity and intracellular putrescine, spermidine, and spermine levels (**Figure 10F**). Notably, exogenous spermidine substantially restored the loss of cell viability induced by CCT6A knockdown (**Figure 10G**). Together, these perturbation, metabolic, and rescue experiments support a functional role for CCT6A in AML cell fitness and indicate that disruption of polyamine homeostasis contributes to the phenotype induced by CCT6A silencing.

## 4 Discussion

Polyamine metabolism emerged as a cell-state-associated feature of AML rather than a uniformly increased metabolic program across the bone marrow compartment. Its activity was preferentially concentrated in LSC-like, AML progenitor, proliferative AML, and blast populations, and within AML blasts was associated with increased stemness, proliferation, and DNA damage response together with reduced myeloid differentiation. This organization is consistent with growing evidence that metabolic dependencies in AML are shaped by developmental state and genomic context rather than being shared equally across leukemic populations (32–35). In particular, spermidine-dependent eIF5A hypusination and KAT7 expression have been implicated in leukemia stem and progenitor cell maintenance (36), while polyamines can couple metabolic demand to translation and epigenetic regulation (6, 13, 37). Our findings extend these observations by placing polyamine metabolism within the cellular hierarchy of AML and suggest that polyamine-active states preferentially accompany immature and proliferative leukemic phenotypes.

Polyamine-active AML states were also associated with substantial changes in the surrounding immune context. High-polyamine cells displayed broader inferred intercellular communication and altered HLA class I- and major histocompatibility complex class II (MHC-II)-related signaling, while patient-level analyses linked adverse risk features to stromal and myeloid enrichment. These observations are biologically plausible because polyamines regulate macrophage activation, tumor-derived N1-acetylspermidine can promote macrophage-mediated immune suppression (38), and polyamine availability can support the survival of immunosuppressive myeloid cells under metabolically adverse conditions (39, 40). Importantly, an immune-inflamed state is not necessarily equivalent to effective antitumor immunity; increased immune signaling may coexist with dysfunctional or suppressive cellular programs. Accordingly, our CellChat, immune-deconvolution, and TIDE results should be interpreted as evidence that polyamine-active AML states coexist with a distinct immune microenvironment, rather than as proof that polyamine metabolism directly drives immune remodeling.

The cellular signal identified by single-cell analysis remained detectable at the patient level, providing an important bridge between metabolic cell state and clinical heterogeneity. Genes selected specifically from polyamine-associated AML blasts separated bulk AML samples into two biologically distinct subtypes: a hypermetabolic/proliferative state characterized by oxidative phosphorylation, fatty-acid metabolism, MYC/E2F activity, and DNA repair, and a stem-like, immune-inflamed state with distinct mutation, cytogenetic, and immune features. The latter was associated with inferior survival in BeatAML, GSE106291, and the pooled cohort, although this relationship was not reproduced in TCGA-AML, indicating that its prognostic effect is cohort dependent. This distinction is important because the value of these subtypes lies primarily in resolving biologically different AML states rather than defining a universally prognostic classification. The RSF risk model provides a complementary representation of this biology: risk scores localized preferentially to leukemic blast and progenitor populations and were associated at the patient level with distinct myeloid/stromal microenvironmental features. Similar studies have shown that transcriptional and inflammatory cell states can provide clinically relevant information beyond conventional genomic classification (20, 23). Thus, the convergence of single-cell states, bulk molecular phenotypes, and survival-associated features supports polyamine-related biology as one dimension of clinically relevant AML heterogeneity, while the molecular subtype and prognostic score capture related but non-identical aspects of that heterogeneity.

CCT6A provides the strongest functional connection between this transcriptomic framework and AML cell behavior. Beyond its high contribution to the prognostic model and dependency signal in AML cell lines, CCT6A was overexpressed in AML bone marrow samples, and its silencing reduced MOLM-13 cell viability while increasing apoptosis, accompanied by decreased PCNA and Bcl-2 and increased Bax expression. More importantly, CCT6A knockdown reduced ODC1 activity together with intracellular putrescine, spermidine, and spermine, directly linking CCT6A perturbation to altered polyamine homeostasis. Exogenous spermidine substantially restored the loss of cell viability caused by CCT6A silencing, indicating that disruption of the polyamine pool contributes to the observed cellular phenotype. This finding is notable because CCT6A is an ATP-dependent TCP-1 ring complex/chaperonin-containing TCP-1 (TRiC/CCT) component (41), and accumulating studies in other cancers have connected CCT6A with proliferation, apoptosis, glycolytic and metabolic reprogramming, MYC- and AKT-related signaling, stemness, chemoresistance, and tumor–microenvironment interactions (42–50). These observations raise the possibility that proteostasis and polyamine metabolism intersect in CCT6A-dependent AML cell fitness. However, the present data do not establish a direct CCT6A–ODC1 interaction, and whether CCT6A affects ODC1 stability, other polyamine-metabolic enzymes, or upstream regulatory pathways remains to be determined.

The functional rescue by spermidine also strengthens the translational relevance of the polyamine axis itself. Experimental disruption of spermidine metabolism has previously been shown to impair AML leukemia stem and progenitor cell function, and recent studies demonstrate that simultaneous inhibition of polyamine biosynthesis and uptake using difluoromethylornithine (DFMO) and the polyamine-transport inhibitor AMXT 1501 suppresses acute leukemia growth and promotes apoptosis in preclinical models (51). Dual inhibition of polyamine synthesis and transport has also entered phase I clinical evaluation in advanced solid tumors (52), establishing the feasibility of pharmacologically depleting the polyamine pool in patients. These observations provide a rationale for testing whether polyamine-active or CCT6A-high AML states identify tumors with enhanced dependence on this metabolic axis. Such a possibility remains to be demonstrated experimentally, however. The predicted drug-sensitivity, gene–chemical, and regulatory-network analyses in the present study should therefore be viewed as hypothesis-generating approaches for therapeutic prioritization rather than evidence of treatment efficacy, and CCT6A is more appropriately regarded at present as a candidate functional dependency than as an established therapeutic target.

Several limitations define the scope of these conclusions. The transcriptomic and clinical analyses were largely retrospective and combined cohorts generated on different platforms and under heterogeneous treatment backgrounds. Although survival associations were reproduced across several datasets, the lack of significant subtype-associated survival separation in TCGA-AML underscores cohort-specific heterogeneity and the need for prospective validation incorporating standardized treatment information and established clinical variables such as European LeukemiaNet (ELN) risk. Likewise, computational estimates of immune infiltration, immunotherapy response, and drug sensitivity require orthogonal experimental validation. At the experimental level, CCT6A perturbation was performed in a single AML cell line, and the clinical expression analysis involved a relatively small sample set; confirmation in additional AML molecular backgrounds, primary leukemia cells, and *in vivo* models, together with pharmacological perturbation of polyamine metabolism and genetic rescue approaches, will therefore be important to further define these metabolic dependencies. Spermidine rescue supports a contribution of altered polyamine homeostasis to the CCT6A-knockdown phenotype but does not define the underlying molecular mechanism. Finally, the spatial transcriptomic analysis was derived from a single AML sample and should be considered exploratory localization evidence rather than independent mechanistic validation.

## 5 Conclusion

Our findings identify polyamine metabolism as a biologically structured feature of AML that preferentially accompanies primitive and proliferative leukemic cell states and extends to patient-level molecular, immune, and prognostic heterogeneity. Integrating these observations across cellular and patient scales led to the prioritization of CCT6A, whose functional perturbation altered AML cell survival, ODC1 activity, and intracellular polyamine homeostasis, with spermidine rescue providing evidence that the polyamine pool contributes to this phenotype. Rather than establishing polyamine metabolism as a direct driver of the immune microenvironment or CCT6A as a validated therapeutic target, these results define a CCT6A–polyamine relationship that provides a biologically coherent basis for further mechanistic investigation and for evaluating polyamine-directed vulnerabilities in AML.

## Data Availability

Publicly available datasets were analyzed in this study. These data can be found in the Gene Expression Omnibus under accession numbers GSE185381, GSE116256, GSE279576, and GSE106291; Zenodo under record 3345981; and the TCGA-AML and BeatAML data portals. The raw data supporting the conclusions of this article will be made available by the authors, without undue reservation.
