# Supplementary material for: Integrative single-cell and bulk transcriptomics define polyamine-associated cell states in acute myeloid leukemia and implicate CCT6A in polyamine homeostasis
Source: Front Immunol. 2026 Sep 8;17:1914578. doi: 10.3389/fimmu.2026.1914578 (PMC13593858; doi:10.3389/fimmu.2026.1914578)
Supplement: Supplementary file 1 [file Table1.docx]

**Supplementary Figure 1. Genomic, clinical, stemness, and immune characteristics of molecular subtypes.**

(A, B) Somatic mutation profiles of Cluster A and Cluster B, respectively.

(C) Differentially mutated genes between the two clusters, with odds ratios and 95% confidence intervals.

(D) Cytogenetic risk distribution in Cluster A and Cluster B.

(E) Immune infiltration profiles estimated using multiple deconvolution algorithms.

(F) CellStat score distributions between the two clusters.

(G) Stemness score distributions between the two clusters.

**Supplementary Figure 2. Risk-associated gene expression patterns and gene set enrichment analysis.**

(A) Heatmap showing genes positively correlated with the risk score.

(B) Heatmap showing genes negatively correlated with the risk score.

(C) GSEA bubble plot showing enriched GO Biological Process gene sets associated with the risk score.

(D) GSEA bubble plot showing enriched KEGG pathways associated with the risk score.

(E) GSEA bubble plot showing enriched Reactome pathways associated with the risk score.

(F) GSEA bubble plot showing enriched Hallmark gene sets associated with the risk score. Positive and negative normalized enrichment scores indicate pathways associated with high- and low-risk features, respectively.

**Supplementary Figure 3. Immune microenvironment profiling and immunotherapy response prediction by risk group.**

(A) Heatmap showing immune cell infiltration abundance between the high- and low-risk groups.

(B) Heatmap showing immune-related gene expression between the high- and low-risk groups.

(C–J) Box plots comparing TIDE CAF score (C), TIDE dysfunction score (D), TIS score (E), IFNG score (F), IPRES score (G), M2 macrophage score (H), MDSC score (I), and immunophenoscore (IPS) (J) between risk groups. Statistical significance was assessed using the Wilcoxon rank-sum test.

(K) Stacked bar plot showing the proportions of TIDE-predicted immunotherapy responders and non-responders in each risk group. Differences in responder proportions were assessed using Fisher’s exact test.

**Supplementary Figure 4. Predicted drug sensitivity analysis by risk group.**

(A) Lollipop chart showing drugs with significantly different predicted IC₅₀ values between the high- and low-risk groups (FDR < 0.05).

(B) Violin plots showing predicted IC₅₀ values for the top nine drugs with the largest differences between risk groups.

(C–H) Scatter plots showing correlations between the risk score and predicted IC₅₀ values for Ulixertinib (C), AMG.319 (D), AZ6102 (E), Fulvestrant (F), MN.64 (G), and Sepantronium bromide (H).

**Supplementary Figure 5. Gene–chemical associations and regulatory network analysis of key genes.**

(A) Star network plot showing gene–chemical associations between key genes and associated chemical compounds.

(B) Heatmap showing drug perturbation-induced expression changes of key genes, with colors representing logFC values.

(C) Circular network plot showing upstream regulatory factors of key genes.

(D) Faceted bar plot showing the top upstream regulators for each key gene, with activation and repression indicated by color.

(E) Network plot showing downstream regulatory targets of key genes.

(F) Faceted bar plot showing the top downstream targets for each key gene, with activation and repression indicated by color.

**Supplementary Figure 6. Exploratory spatial transcriptomic localization of AML blasts and key genes.**

(A) Spatial maps showing RCTD-deconvolved cell-type proportions in AML sample GSM8576303_BM2.

(B) Spatial distribution of AML blast proportions, with high-enrichment regions defined as spots above the 75th percentile.

(C) Spearman correlations between AML blast proportions and the abundances of other cell types.

(D) Spearman correlations between AML blast proportions and key gene expression.

(E) Cell type enrichment associated with ENO1 high-expression regions.

(F) Cell type enrichment associated with PRDX5 high-expression regions.
